# Supplementary material for: Association between enrolment with a Primary Health Care provider and amenable mortality: A national population-based analysis in Aotearoa New Zealand
Source: PLoS One. 2023 Feb 3;18(2):e0281163. doi: 10.1371/journal.pone.0281163 (PMC9897554; doi:10.1371/journal.pone.0281163)
Supplement: S1 Appendix — (DOCX) [file pone.0281163.s001.docx]

**S1 Appendix: List of amenable conditions, Ministry of Health 2018**

| **Group** | **Condition** | **ICD-10-AM-II (2012 definition - used from 2000-2009)** | **ICD-10-AM-II (2016 definition - used from 2010 onwards)** | **Notes** |
| --- | --- | --- | --- | --- |
| **Infections** | Pulmonary tuberculosis | A15-A16 | A15-A16 |  |
|  | Meningococcal disease | A39 | A39 |  |
|  | Pneumococcal disease | A40.3, G00.1, J13 | A40.3, G00.1, J13 |  |
|  | Hepatitis C virus |  | B17.1, B18.2 | New in 2016 version |
|  | HIV/AIDS | B20-B24 | B20-B24 |  |
| **Cancers** | Stomach cancer | C16 | C16 |  |
|  | Rectal cancer | C19-C21 | C19-C21 |  |
|  | Bone and cartilage cancer | C40-C41 | C40-C41 |  |
|  | Melanoma of skin | C43 | C43 |  |
|  | Female breast cancer | C50 | C50 | Females only |
|  | Cervical cancer | C53 | C53 |  |
|  | Uterine cancer |  | C54-C55 | New in 2016 version |
|  | Prostate cancer | C61 | C61 |  |
|  | Testis cancer | C62 | C62 |  |
|  | Thyroid cancer | C73 | C73 |  |
|  | Hodgkin lymphoma | C81 | C81 |  |
|  | Acute lymphoblastic leukaemia | C91.0 | C91.0 | ages 0-44 |
| **Maternal and infant disorders** | Complications of pregnancy | O00-O96, O98-O99 | O00-O96, O98-O99 |  |
|  | Complications of the perinatal period | P01-P03, P05-P94 | P01-P03, P05-P94 |  |
|  | Cardiac septal defect | Q21 | Q21 |  |
| **Cardiovascular disorders and diabetes** | Diabetes | E10-E14 | E10-E14 |  |
|  | Valvular heart disease | I01, I05-I09, I33-I37 | I01, I05-I09, I33-I37 |  |
|  | Hypertensive diseases | I10-I13 | I10-I13 |  |
|  | Coronary disease | I20-I25 | I20-I25 |  |
|  | Pulmonary embolism | I26 | I26 |  |
|  | Atrial fibrillation and flutter |  | I48 | New in 2016 version |
|  | Heart failure | I50 | I50 |  |
|  | Cerebrovascular diseases | I60-I69 | I60-I69 |  |
| **Other chronic disorders** | COPD | J40-J44 | J40-J44 |  |
|  | Asthma | J45-J46 | J45-J46 |  |
|  | Peptic ulcer disease | K25-K27 | K25-K27 |  |
|  | Cholelithiasis | K80 | K80 |  |
|  | Renal failure | N17-N19 | N17-N19 |  |
| **Injuries** | Land transport accidents excluding trains | V01-V04, V06-V14, V16-V24, V26-V34, V36-V44, V46-V54, V56-V64, V66-V74, V76-V79, V80.0-V80.5, V80.7-V80.9, V82-V86, V87.0-V87.5, V87.7-V87.9, V88.0-V88.5, V88.7-V88.9, V89, V98-V99 | V01-V04, V06-V14, V16-V24, V26-V34, V36-V44, V46-V54, V56-V64, V66-V74, V76-V79, V80.0-V80.5, V80.7-V80.9, V82-V86, V87.0-V87.5, V87.7-V87.9, V88.0-V88.5, V88.7-V88.9, V89, V98-V99 | Include V00 if using ICD-10-AM-VI |
|  | Accidental falls on the same level | W00-W08, W18 | W00-W08, W18 |  |
|  | Fire | X00-X09 | X00-X09 |  |
|  | Suicide | X60-X84 | X60-X84 |  |
|  | Treatment injury | Y60-Y82 | Y60-Y82 |  |
