# Supplementary material for: Association between enrolment with a Primary Health Care provider and amenable mortality: A national population-based analysis in Aotearoa New Zealand
Source: PLoS One. 2023 Feb 3;18(2):e0281163. doi: 10.1371/journal.pone.0281163 (PMC9897554; doi:10.1371/journal.pone.0281163)
Supplement: S2 Appendix — (DOCX) [file pone.0281163.s002.docx]

| **S2 Appendix:** Socio-demographic characteristics of amenable mortality cases by cause of death | | | | | | | | | | | | | | | | | | | | | |
| --- | --- | --- | --- | --- | --- | --- | --- | --- | --- | --- | --- | --- | --- | --- | --- | --- | --- | --- | --- | --- | --- |
|  | **Cancer** | | **Cardio-Vascular diseases** | | **Infection** | | **Injuries** | | | | **Maternal and Infant Deaths** | | | | **Others** | | | | **Total amenable deaths** | | |
|  | **N** | **Row %** | **N** | **Row %** | **N** | **Row %** | **N** | | **Row %** | | **N** | | **Row %** | | **N** | | **Row %** | | **N** | **Col %** | |
| **Total** | **13,120** | **23.3** | **25,581** | **45.3** | **464** | **0.8** | **8,469** | | **15.0** | | **2,947** | | **5.2** | | **5,833** | | **10.3** | | **56,414** | **100.0** | |
| **Sex** |  |  |  |  |  |  |  | |  | |  | |  | |  | |  | |  |  | |
| Female | 7,343 | 32.4 | 8,504 | 37.5 | 153 | 0.7 | 2,283 | | 10.1 | | 1,381 | | 6.1 | | 3,016 | | 13.3 | | 22,680 | 40.2 | |
| Male | 5,777 | 17.1 | 17,077 | 50.6 | 311 | 0.9 | 6,186 | | 18.3 | | 1,552 | | 4.6 | | 2,817 | | 8.4 | | 33,720 | 59.8 | |
| Unidentified | 0 | 0.0 | 0 | 0.0 | 0 | 0.0 | 0 | | 0.0 | | 14 | | 100.0 | | 0 | | 0.0 | | 14 | 0.0 | |
| **Age (years)** |  |  |  |  |  |  |  | |  | |  | |  | |  | |  | |  |  | |
| Under 1 | 0 | 0.0 | 5 | 0.2 | 19 | 0.7 | 20 | | 0.7 | | 2,800 | | 98.5 | | 0 | | 0.0 | | 2,844 | 5.0 | |
| 01-04 | 2 | 1.6 | 2 | 1.6 | 20 | 16.0 | 77 | | 61.6 | | 12 | | 9.6 | | 12 | | 9.6 | | 125 | 0.2 | |
| 05-14 | 14 | 5.6 | 15 | 6.0 | 2 | 0.8 | 194 | | 77.9 | | 4 | | 1.6 | | 20 | | 8.0 | | 249 | 0.4 | |
| 15-24 | 87 | 3.9 | 69 | 3.1 | 15 | 0.7 | 2,022 | | 90.6 | | 16 | | 0.7 | | 22 | | 1.0 | | 2,231 | 4.0 | |
| 25-44 | 1,179 | 21.6 | 1,258 | 23.0 | 53 | 1.0 | 2,770 | | 50.8 | | 74 | | 1.4 | | 124 | | 2.3 | | 5,458 | 9.7 | |
| 45-64 | 6,048 | 28.1 | 10,703 | 49.7 | 274 | 1.3 | 2,613 | | 12.1 | | 27 | | 0.1 | | 1,869 | | 8.7 | | 21,534 | 38.2 | |
| 65-74 | 5,790 | 24.2 | 13,529 | 56.4 | 81 | 0.3 | 773 | | 3.2 | | 14 | | 0.1 | | 3,786 | | 15.8 | | 23,973 | 42.5 | |
| **Prioritised Ethnicity** |  |  |  |  |  |  |  | |  | |  | |  | |  | |  | |  |  | |
| Māori | 1,797 | 14.5 | 6,241 | 50.2 | 88 | 0.7 | 2,016 | | 16.2 | | 849 | | 6.8 | | 1,441 | | 11.6 | | 12,432 | 22.0 | |
| Pacific people | 849 | 18.2 | 2,668 | 57.1 | 29 | 0.6 | 423 | | 9.1 | | 424 | | 9.1 | | 281 | | 6.0 | | 4,674 | 8.3 | |
| NMNP^1^ | 10,474 | 26.6 | 16,672 | 42.4 | 347 | 0.9 | 6,030 | | 15.3 | | 1,674 | | 4.3 | | 4,111 | | 10.5 | | 39,308 | 69.7 | |
| **Area level deprivation (quintiles)** | | | | | | | |  | |  | |  | |  | |  | |  |  | |  |
| Least deprived | 1,679 | 33.1 | 1,862 | 36.7 | 45 | 0.9 | 871 | | 17.2 | | 261 | | 5.1 | | 360 | | 7.1 | | 5,078 | 9.0 | |
| 2 | 3,091 | 27.2 | 4,748 | 41.8 | 92 | 0.8 | 1,898 | | 16.7 | | 512 | | 4.5 | | 1,008 | | 8.9 | | 11,349 | 20.1 | |
| 3 | 3,890 | 23.7 | 7,297 | 44.4 | 155 | 0.9 | 2,510 | | 15.3 | | 792 | | 4.8 | | 1,793 | | 10.9 | | 16,437 | 29.1 | |
| 4 | 3,086 | 19.4 | 7,842 | 49.2 | 112 | 0.7 | 2,107 | | 13.2 | | 833 | | 5.2 | | 1,955 | | 12.3 | | 15,935 | 28.2 | |
| Most deprived | 819 | 16.2 | 2,660 | 52.7 | 41 | 0.8 | 607 | | 12.0 | | 392 | | 7.8 | | 530 | | 10.5 | | 5,049 | 8.9 | |
| Missing | 555 | 21.6 | 1,172 | 45.7 | 19 | 0.7 | 476 | | 18.6 | | 157 | | 6.1 | | 187 | | 7.3 | | 2,566 | 4.5 | |
| **Total** | **13,120** | **23.3** | **25,581** | **45.3** | **464** | **0.8** | **8,469** | | **15.0** | | **2,947** | | **5.2** | | **5,833** | | **10.3** | | **56,414** | **100.0** | |

**Note:** 1) NMNP is the Non-Māori, Non-Pacific population
