# Supplementary material for: Association between enrolment with a Primary Health Care provider and amenable mortality: A national population-based analysis in Aotearoa New Zealand
Source: PLoS One. 2023 Feb 3;18(2):e0281163. doi: 10.1371/journal.pone.0281163 (PMC9897554; doi:10.1371/journal.pone.0281163)
Supplement: S3 Appendix — (DOCX) [file pone.0281163.s003.docx]

| **S3 Appendix: Socio-demographic characteristics of the study population by enrolment status at death (premature deaths), 2008-2017** | | | | | |
| --- | --- | --- | --- | --- | --- |
|  | **Enrolled at Death** | | **Not-enrolled at Death** | | **Chi squared***  **(p value)** |
|  | **N** | **%** | **N** | **%** |  |
| **Sex** |  |  |  |  |  |
| Female | 43941 | 41.8 | 5040 | 39.5 | 0.001 |
| Male | 61294 | 58.2 | 7716 | 60.5 |  |
| **Age (years)** |  |  |  |  |  |
| Under 1 | 559 | 0.5 | 6,747 | 52.9 |  |
| 01-04 | 560 | 0.5 | 29 | 0.2 | 0.001 |
| 05-14 | 653 | 0.6 | 35 | 0.3 |  |
| 15-24 | 2,947 | 2.8 | 583 | 4.6 |  |
| 25-44 | 9,153 | 8.7 | 1,141 | 8.9 |  |
| 45-64 | 42,455 | 40.3 | 2,496 | 19.6 |  |
| 65-74 | 48,908 | 46.5 | 1,725 | 13.5 |  |
| **Ethnicity** |  |  |  |  |  |
| Māori | 20,996 | 20.0 | 3,186 | 25.0 | 0.001 |
| Pacific people | 6,891 | 6.5 | 1,824 | 14.3 |  |
| Non-Māori Non-Pacific | 77,348 | 73.5 | 7,746 | 60.7 |  |
| **Area level deprivation (quintiles)** | |  |  |  |  |
| Least deprived | 10,172 | 9.7 | 1,001 | 7.8 | 0.001 |
| 2 | 22,541 | 21.4 | 2,136 | 16.7 |  |
| 3 | 31,389 | 29.8 | 3,272 | 25.7 |  |
| 4 | 29,013 | 27.6 | 3,314 | 26.0 |  |
| Most deprived | 8,396 | 8.0 | 1,406 | 11.0 |  |
| Missing | 3,724 | 3.5 | 1,627 | 12.8 |  |
| **Total** | **105,235** | 100.0 | **12,756** | 100.0 |  |
| **Note:** NMNP = Non-Māori Non-Pacific; Enrolled includes those having at least one active enrolment status during the study years; Not-enrolled includes those having no PHO record or having no active enrolment status during the study years.  * Chi squared test of association between the enrolment status and the population sub-groups (characteristics). | | | | | |
