# Supplementary material for: Association between enrolment with a Primary Health Care provider and amenable mortality: A national population-based analysis in Aotearoa New Zealand
Source: PLoS One. 2023 Feb 3;18(2):e0281163. doi: 10.1371/journal.pone.0281163 (PMC9897554; doi:10.1371/journal.pone.0281163)
Supplement: Attachment — Submitted filename: Response to Reviewers.docx [file pone.0281163.s004.docx]

Thank you so much for your valuable comments and suggestions. Please find our responses below – *blue italicized* texts. We have tried to make the responses concise. Please let us know should you require any further clarification. We would be happy to work further and respond accordingly.

**Note:** The page numbers and line numbers on the responses are according to that in the track-changed document uploaded into the portal.

PONE-D-22-19204

Association between enrolment with a Primary Health Care provider and amenable mortality: A population-based analysis in Aotearoa New Zealand

PLOS ONE

**Editor’s comments:**

The description of the characteristics of the population not enrolled is a relevant topic in the manuscript. It is presented as a purpose of the study in the abstract. Also, in the discussion, the authors report inequities in enrolment by ethnicity. However, in results section, there is not a clear description of the association of enrolment with ethnicity and other variables.[see comment under R2C2]

*Added line numbers 225-230, page 10 – “The enrolled and not enrolled are near equally likely to be male although males dominate in both groups (approx. 59%). The not-enrolled are more likely to be under 1 (62% vs .5%) and less likely to be over 45 (26% vs 86%) compared to the enrolled. In comparison, the not enrolled are more likely to be Māori and Pacific peoples but the Non-Māori, Non-Pacific peoples still make up the majority of the deaths (61%). The not enrolled are more likely to be in the most deprived category (11% vs 8%) while the enrolled are more likely to be in the lesser deprived categories. (Table 2).”*

Besides, the association between enrolment and amenable mortality, stratified by age, needs further description. The more significant differences (OR=4.9) are observed under 1 year of age. As expected, the most common causes of death in such group are related to perinatal conditions (Appendix 2). It seems likely that most of such newborns died in hospitals without any contact with primary care, and the enrolment data correspond to their mothers. Please, clarify this point.

*We agree this, and have added a sentence to the discussion (see line 341-342, Page 15)*

*The rationale for the age-stratified analysis is provided in page 12, lines 308-312.*

Also, ethnicity and socioeconomic groups diverge in the causes of amenable deaths. There are noticeable differences in “Maternal and Infant Deaths” (almost limited to the perinatal ones). In my view, these findings could be developed.

*Added lines 212-217, page 10. The details of the amenable deaths in terms of the major primary cause categories are provided in Appendix 2. The cardiovascular diseases category represents almost half (45.3%) of the total amenable deaths followed by cancers (23.3%), injuries (15.0%), maternal and infant disorders (5.2%) and infections (0.8%). Māori represents 22.0% in the total amenable deaths. In comparison to other ethnicity groups, they died more from Injuries (16.2% versus 9.1% among Pacific peoples and 15.3% NMNP) and least from Cancers (14.5% versus 18.2% among Pacific peoples and 26.6% among NMNP) . Similarly, the causes of amenable deaths also vary across the socio-economic deprivation groups, with those living in the least deprived areas representing highest proportion of Cancers deaths (33.1%) and lowest proportion of the cardiovascular deaths (36.7%).*

**Reviewer #1:**

1)

In the abstract you state: We examine the characteristics of populations not enrolled, and whether enrolment is associated with amenable mortality.
In the introduction you write: The purpose of this analysis is to quantify the impact of not being enrolled with a PHO in Aotearoa New Zealand on amenable mortality, i.e. mortality for conditions that are amenable to health care interventions
And I think the tables do answer these aims.
Table 1: Socio-demographic characteristics of the study population by premature and amenable death status
Table 2: Socio-demographic characteristics of the study population by enrolment status
Table 3: Socio-demographic characteristics of amenable mortality cases and ORs of amenable deaths versus nonamenable deaths
However, I am not sure whether we need the detail on premature mortality in the paper, if you are not going to model this. But this is OK. I am more interested in why you didn’t model the factors associated with enrolment. In the discussion you talk about the groups who are less likely to be enrolled, and I am really interested in whether there are interactions between age, sex and socio-economic status and ethnicity. It is OK that you didn’t do this, but I wonder whether it would make some of your discussion stronger.

*The statistics on premature mortality are national statistics which makes them important for that reason.*

*Analysing interactions with age group and with ethnicity might be problematic due to numbers.*

2)

Table 4 – I am not clear what you have done. Have you run separate models for each age group, or used an interaction term. I have understood the OR to be for ‘enrolled V not-unrolled’.

I am concerned that the enrolled V not enrolled in the youngest age group being associated with mortality may be complicated by babies dying before they have a chance to be enrolled. Have you thought about this?

*First part of the question: Each age group was modelled separately. It has now been made clear in the text in page 13, Table 4 Note.*

*Second part of the question: We agree this, and have added a sentence to the discussion (see line 405-406, Page 15)*

3)

In the discussion you say:
This could explain in part the differential results that we see by ethnicity, where Māori are less likely to enrol with a PHC provider, and experience higher rates of amenable mortality.
I am not sure why you don’t include/separately discuss Pacific peoples in the section, who are also more likely to die prematurely, and their death classed as amenable.

*We focus here on Māori, which is because of the Treaty of Waitangi obligation. Article Three of the treaty says – the Crown promised that its obligations to New Zealand citizens are owed equally to Māori. [https://dpmc.govt.nz/our-programmes/policy-project/policy-methods-toolbox/treaty-waitangi-analysis]*

4)

In the discussion, I think you should highlight that the definition of amenable might be a limitation, it is a govt definition, so fine to use, but presumably not everyone would agree. I am sure some people might argue that some of the conditions are more affected by socio-economic factors and not healthcare.

*Added a limitation: page 16, line 418-419: Furthermore, some of the conditions included in the definition of amenable mortality could relate more to socio-economic factors than healthcare (Mackenbach et al., 2013).*

Minor Comments
5)

Methods – The sentence about merging European and Asian was difficult to understand, I think you merged the two groups because the Asian group was small.

*Added: “Due to small sample sizes of the Asian category in our study, particularly for the unenrolled cases, the latter two categories were combined into a Non-Māori, Non-Pacific (NMNP) group”, page 6, lines 154-156.*

6)

Table 1 has a footnote 3 and I am not sure that I could find it in the table.

*The table has been redone as suggested by Reviewer 2 and the original been replaced.*

7)

Table 2 – can you make foot note 4 column percent by group? Or something equivalent

*The table has been redone as suggested by Reviewer 2 and the original has been replaced.*

8)

Please look at the heading and subject matter of this paragraph – it doesn’t seem match.
Relationship between primary health care enrolment and premature mortality
The adjusted odds of amenable mortality among those not enroled are 38.5% higher [OR=1.38, 95% CI 1.30-1.47] than those enrolled (Table 3). Similarly, males [OR 1.1, 95% CI 1.07-1.12] compared to females, youths aged 15-24 years [OR 6.37, 95% CI 5.18-7.88] compared to those aged 01-04 years, Pacific People and Māori [OR 1.32, 95% CI 1.25-1.38 and 1.22, 1.19-1.26 respectively] compared to nonMāori, non-Pacific people, and those living in the most deprived areas [OR=1.12, 95% CI 1.06-1.19] compared to those in the least deprived areas are more likely to die from amenable causes.

*Paragraph heading changed to “Relationship between primary health care enrolment and amenable mortality”*

9)

Can you clarify whether only the under 75 deaths were included in the amenable V non-amenable models? I think you may have said, but couldn’t find.

*Sixth line under “Socio-demographic variables” section and second line under “Outcome variables” section. It’s also mentioned in the abstract.*

10)
Apologies, but I had never seen ‘Aotearoa New Zealand’ before. I therefore did not realise it was a national study. I am not sure of the best approach to this for a less informed audience. In the title could the word national be inserted before Aotearoa New Zealand?

*Added ‘national’ on the title as “Association between enrolment with a Primary Health Care provider and amenable mortality: A national, population-based analysis in Aotearoa New Zealand”.*

Reviewer #2: 1. Causal language
“The purpose of this analysis is to quantify the impact of not being enrolled with a PHO in Aotearoa New Zealand on amenable mortality”
“This confirms our initial hypothesis that not being enrolled and, thus, missing out on the benefits
that enrolling provides, will impact negatively on health outcomes.”
I suggests authors refrain from using the language that implies causality in the association between PHC enrollment and amenable mortality.

*Thanks, changed in the main document now:*

*line 78-79, page 4: “The purpose of this analysis is to quantify the association of not being enrolled with a PHO in Aotearoa New Zealand on amenable mortality, i.e. mortality for conditions that are amenable to health care interventions”.*

*Lines 333-334, page 13: “This analysis is in line with our initial hypothesis that not being enrolled and, thus, missing out on the benefits that enrolling provides, is associated with negative health outcomes”.*

2. Descriptive statistics tables in the manuscript itself and in the appendix to the manuscript depict row statistics. This makes it much harder to a reader to understand how characteristics of the enrolled and not enrolled subsamples differ. I suggest authors convert tables to column percentages everywhere not just for “total” column.

*Thank you so much. Table 1, Table 2 and Appendix 3 Table are changed as per your suggestions and the descriptions in the main text are all updated accordingly.*

3. Stemming from point 2 above the section of the paper that should have described how demographic characteristics of the enrolled subsample differ from those of not enrolled subsample is murky. There is chi square test present in Table 2 but it’s unclear what this test actually compares to what. Manuscript needs a clear section that would describe how gender, age, ethnicity and area deprivation composition of the enrolled subsample differs from not enrolled.

*The table has been redone as suggested by Reviewer 2 and the original been replaced. A notation has been added in the tables for the chi-squared test: “*Chi squared test of association between the enrolment status and the population sub-groups (characteristics)”.*

4. Manuscript simply presents adjusted associations between PHC enrollment and amenable mortality. The authors never make a case why these estimates should be interpreted as causal while the language of the paper suggests it should. Authors should either show why it’s appropriate to interpret their estimates as causal or admit that the estimates are not causal.

*Thanks, we have changed the language to imply ‘association’ rather than causality.*

5. Table 2 shows that association between not enrolled and amenable mortality is statistically significant but the sign of this association in the bivariate and adjusted models are opposite of one another. Why is that?

*We did talk about our exploration of this in the paragraph: lines 308-312 on page 12, after Table 3.*

*“The OR for the enrolment variable changed from 0.94 in the bivariate model to 1.38 in the multi-variate model. To explore which variable caused this change in size of the OR, a model with mortality and enrolment status was fitted with each of the socio-demographic variables in turn. This showed that age group was the variable that affected this change moving the OR from 0.94 to 1.34 while for the other variables the change was less than 0.1.”*

*The implication is that the age structure of the two groups differ and adjusting for that changes the ORs.*

6. According to table 3, 15-25 year old adolescents and young adults are likely to die from amenable causes than 5-14 year old children. According to table 4, 15-24 year old adolescents and young adults who are not enrolled are less likely to die from amenable causes. Could authors discuss this result? Since authors interpret results as causal would they suggest that disenrolling youth starting age 15 from primary health care provider would save their lives?

*We did talk about this in this paragraph: lines 334 - 336, page 15.*

*“For young people, not being enrolled is protective when compared to those who are enrolled*

*(see table 4). However, being enrolled shows a demonstrated health need and it may be that the enrolled group have lower health status, on average. It is likely that unenrolled young people would acquire health benefits from enrolling.”*

*We have added these examples of benefits, lines 411 - 415, page 15.*

*There is likely “adverse selection” going on. Ill young people do enrol to get health care but apparently-healthy young people don’t as they perceive they don’t need health care. Young people would benefit from enrolling, even if well, because of testing for unobserved diseases, reduced price contraceptives, direction to secondary and maternal services etc.*

Mackenbach, J. P., Hoffmann, R., Khoshaba, B., Plug, I., Rey, G., Westerling, R., . . . McKee, M. (2013). Using 'amenable mortality' as indicator of healthcare effectiveness in international comparisons: Results of a validation study. *Journal of Epidemiology and Community Health, 67*(2), 139-146. doi:10.1136/jech-2012-201471
